# PCSK9 inhibition in high-risk patients

**DOI:** 10.18632/aging.102621

**Published:** 2019-12-11

**Authors:** Laurien E. Zijlstra, Simon P. Mooijaart, J. Wouter Jukema

**Affiliations:** 1 Department of Cardiology Leiden University Medical Center, The Netherlands; 2 Department of Gerontology and Geriatrics Leiden University Medical Center, The Netherlands; 3 Institute for Evidence-based Medicine in Old Age (IEMO), Leiden, the Netherlands

**Keywords:** older patients, cardiovascular disease, lipid-lowering, PCSK9 inhibiting, plaque burden

Cardiovascular disease (CVD) burden is increasing with advancing age, resulting in high mortality and morbidity worldwide. As societies continue to age, >80% of individuals dying from CVD are 65 years or older. Main reason for increased CVD in older people is an absolute increase in atherosclerotic plaque burden [1]. Patients with high plaque burden often have highest clinical benefit from cardiovascular treatment: because absolute risk is high, absolute risk reductions are also relatively high. Therefore, it remains a key point to identify these the subsets of patients with highest plaque burden to provide an optimal treatment strategy with high benefit but low risk. Measuring total plaque burden could provide the most accurate risk stratification. But although various imaging techniques exist, none are currently suitable to implement in routine daily practice. Therefore, surrogate markers of plaque burden should be used, as low-density lipoprotein cholesterol (LDL-C). In addition, patients can be classified according to clinical features that also reflect plaque burden, which provides an easy and cost-effective manner to achieve optimal treatment. Well-known clinical high risk features include patients with chronic kidney disease or diabetes, and also patients with known vascular disease such as a history of coronary artery bypass grafting (CABG) or atherosclerosis in multiple vascular beds (polyvascular disease).

Relatively frail patients are more prone to side effects of treatment, for instance due to an increased bleeding risk. Therefore, finding treatment for primary or secondary prevention with high benefit but low risk of adverse effects is important, especially in older patients. Standard cardiovascular treatment options include medication as aspirin or specific oral anticoagulants, beta-blockers, antihypertensives and lipid-lowering, next to life-style modification, e.g. smoking cessation and regular exercise. Lipid-lowering provides plaque stability and is relatively safe, as the most clinically relevant adverse effect of statins is myopathy. However, especially in older patients statin-associated muscle symptoms can be problematic in daily life. The available evidence from trials indicates that statin therapy produces significant reductions in major adverse cardiovascular events (MACE) irrespective of age, although evidence indicates there is no benefit among patients aged >75 years who do not already have evidence of occlusive vascular disease. Accordingly, international guidelines recommend statin treatment for patients with established cardiovascular disease as secondary prevention for older people in the same way as for younger patients [2]. However, two other key points in optimal treatment for older patients should be noted. First, life-expectancy should be taken into account depending on the lag time to benefit of treatment. Second, expending life-expectancy is only of interest if quality of life remains acceptable.

Relatively new lipid-lowering drugs are PCSK9 (proprotein convertase subtilisin–kexin type 9) inhibitors. PCSK9 inhibiting provides the opportunity to reduce LDL-C to less than levels achievable with statins in most patients and is therefore a therapeutic option for high-risk patients, or for patients in which current treatment is insufficient due to inadequate effect or intolerance for statins. The ODYSSEY OUTCOMES trial showed that MACE were reduced with the PCSK9 inhibitor alirocumab compared with placebo in 18,924 patients with recent acute coronary syndrome (ACS) and elevated atherogenic lipoproteins despite intensive statin therapy (hazard ratio [HR] of 0.85; 95% confidence interval [CI], 0.78 to 0.93; P<0.001). Furthermore, three recent subanalyses of ODYSSEY OUTCOMES showed high risks of MACE with large absolute reductions in those risks with alirocumab therapy in patients with clinically identifiable high plaque burden, including patients with a history of CABG, diabetes and polyvascular disease [3–5].

Although ODYSSEY OUTCOMES was not specifically designed for the older population, a subanalysis showed that the beneficial effect of alirocumab was independent of age and without significant safety issues in the 5084 (26.9%) older patients ≥65 years [6]. Of note, only 1007 (5.3%) patients were ≥75 years and 42 (0.2%) ≥85 years, limiting the power to detect differences in these subgroups. Another recent subanalysis of ODYSSEY OUTCOMES showed that alirocumab decreased the risk of any stroke with a hazard ratio (HR) of 0.72 (95% CI 0.57 to 0.91) and ischemic stroke [0.73 (0.57 to 0.93)] without increasing hemorrhagic stroke [0.83 (0.42 to 1.65)] [7]. As primary treatment goal in older patients should be maintaining or improving quality of life, prevention of strokes is of utmost importance, as stroke can lead to limitations in functional capacity and cognitive function, leading to a significant reduction in quality of life.

In conclusion, it is important to identify subsets of patients for optimal treatment strategies in atherosclerosis, so that efficacy and efficiency are optimized. Monitoring true plaque burden would probably provide the most accurate mechanistic stratification of vascular risk. However, this is clinically not yet feasible in routine practice, in contrast to identifying patients based on easily identifiable risk factors as surrogate plaque marker. Among other established and evolving therapies in atherosclerosis, treatment with PCSK9 inhibitors has high clinical benefit but with few side effects and is therefore potentially suitable also for older patients. Calendar age per se is not a contra-indication for PCSK9 inhibitors, however, the importance of biological age, geriatric impairments and frailty remains to be studied [8].
